# Monasone Naphthoquinone Biosynthesis and Resistance in *Monascus* Fungi

**DOI:** 10.1128/mBio.02676-19

**Published:** 2020-02-04

**Authors:** Mu Li, Lijing Kang, Xiaoli Ding, Jiao Liu, Qingpei Liu, Yanchun Shao, István Molnár, Fusheng Chen

**Affiliations:** a Hubei International Scientific and Technological Cooperation Base of Traditional Fermented Foods, Key Laboratory of Environment Correlative Dietology, College of Food Science and Technology, Huazhong Agricultural University, Wuhan, Hubei, China; b Southwest Center for Natural Products Research, College of Agriculture and Life Sciences, University of Arizona, Tucson, Arizona, USA; Cornell University

**Keywords:** *Monascus* spp., naphthoquinone, nested biosynthetic pathway, supercluster, resistance mechanism

## Abstract

Despite the important biological activities of natural product naphthoquinones, the biosynthetic pathways of and resistance mechanisms against such compounds remain poorly understood in fungi. Here, we report that the genes responsible for the biosynthesis of *Monascus* naphthoquinones (monasones) reside within the gene cluster for *Monascus* azaphilone pigments (MonAzPs). We elucidate the biosynthetic pathway of monasones by a combination of comparative genome analysis, gene knockouts, heterologous coexpression, and *in vivo* and *in vitro* enzymatic reactions to show that this pathway branches from the first polyketide intermediate of MonAzPs. Furthermore, we propose that the monasone subset of biosynthetic genes also encodes a two-tiered resistance strategy in which an inducible monasone-specific exporter expels monasones from the mycelia, while residual intracellular monasones may be rendered nontoxic through a multistep reduction cascade.

## Abstract (precis)

The genes for *Monascus* naphthoquinone (monasone) biosynthesis are embedded in and form a composite supercluster with the *Monascus* azaphilone pigment biosynthetic gene cluster. Early biosynthetic intermediates are shared by the two pathways. Some enzymes encoded by the supercluster play double duty in contributing to both pathways, while others are specific for one or the other pathway. The monasone subcluster is independently regulated and inducible by elicitation with competing microorganisms. This study illustrates genomic and biosynthetic parsimony in fungi and proposes a potential path for the evolution of the mosaic-like azaphilone-naphthoquinone supercluster. The monasone subcluster also encodes a two-tiered self-resistance mechanism that models resistance determinants that may transfer to target microorganisms or emerge in cancer cells in case of naphthoquinone-type cytotoxic agents.

## INTRODUCTION

Quinones, including benzo-, naphtho-, phenanthra-, and anthraquinones, are widely distributed and play divergent roles in animals, plants, and microorganisms (1, 2). Among quinones, naphthoquinones are especially important as secondary metabolites (SMs) or their precursors in fungi (3). To date, more than 100 naphthoquinones with various structures and physicochemical properties have been identified from more than 60 filamentous fungi (4–6). These compounds display various biological activities such as antibacterial, antifungal, anti-inflammatory, and anticancer activities due to their propensity to inhibit respiration and damage DNA (5, 7). Accordingly, several drugs with a naphthoquinone pharmacophore have been developed and marketed, such as doxorubicin against metastatic cancers, idarubicin against acute myeloid leukemia, mitoxantrone against prostate cancer and multiple sclerosis, and vitamin K against hemorrhagic disease (8, 9).

Naphthoquinones have also been considered model compounds to study polyketide biosynthesis in filamentous fungi because of their wide distribution and various bioactivities (6, 10). Unexpectedly, these studies revealed significant differences in naphthoquinone biosynthesis, indicating that the corresponding pathways are polyphyletic (6, 7, 10).

Fungi also need to develop self-protection strategies to avoid committing suicide upon the production of bioactive SMs; thus, fungal SM biosynthetic gene clusters routinely encode one or more self-resistance determinants (11–13). Importantly, the genes encoding such self-resistance mechanisms serve as one of the major pools of preexisting antimicrobial resistance for horizontal gene transfer into clinically important microorganisms (12). The same self-resistance determinants also serve as models and predictors for the various mechanisms by which human cells develop resistance to cytotoxic drugs (8, 13). Thus, elucidation of self-resistance mechanisms not only unmasks basic cellular processes in the producing fungi, but also reveals possible avenues of resistance against applied therapeutics (4, 11). Unfortunately, only a few studies have addressed resistance against naphthoquinones. While these studies concentrated on enzymatic degradation by reductases (14), it is still unclear if other naphthoquinone resistance determinants exist in fungi.

Recently, we found that 1,4-naphthoquinone-based natural product congeners with antimicrobial activities cooccur with *Monascus* azaphilone pigments (MonAzPs). MonAzPs have been used extensively as natural food coloring agents for more than two thousand years, and we and others have intensively investigated their biosynthesis in previous studies (15–17). The 1,4-naphthoquinone coproducts, hereinafter called monasones (*Monascus* naphthoquinones), are detectable only in trace amounts in MonAzP fermentations of wild-type isolates of *Monascus* spp. (15, 16). However, monasones are produced in substantial amounts by various mutants with gene knockouts in the MonAzP biosynthetic gene cluster (MABGC) (15–17). Nevertheless, the biosynthetic steps that yield these naphthoquinone polyketides and the self-resistance mechanisms of the producer fungus against these antimicrobial products remain uncharacterized (15–17).

In this study, we first prove that monasones are bona fide antimicrobial SMs produced by Monascus ruber M7 upon elicitation by competing microorganisms. Then, we elucidate the biosynthesis of monasones and show that the relevant enzymes are encoded within an independently regulated subset of the MABGC. We also show that this subset of genes may endow self-resistance against monasones through detoxification and specific efflux. Our results reveal an unexpected mechanism by which filamentous fungi repurpose pluripotent early SM intermediates and flexible SM biosynthetic enzymes to produce structurally and functionally distinct SMs under different physiological and ecological conditions. We also show that the biosynthesis of these distinct metabolites is encoded in a nested, composite biosynthetic gene supercluster that imparts multilevel synergistic resistance against the potential self-harm caused by these compounds.

## RESULTS

### Monasones are bona fide SMs of *M. ruber* M7.

**FIG 1:**
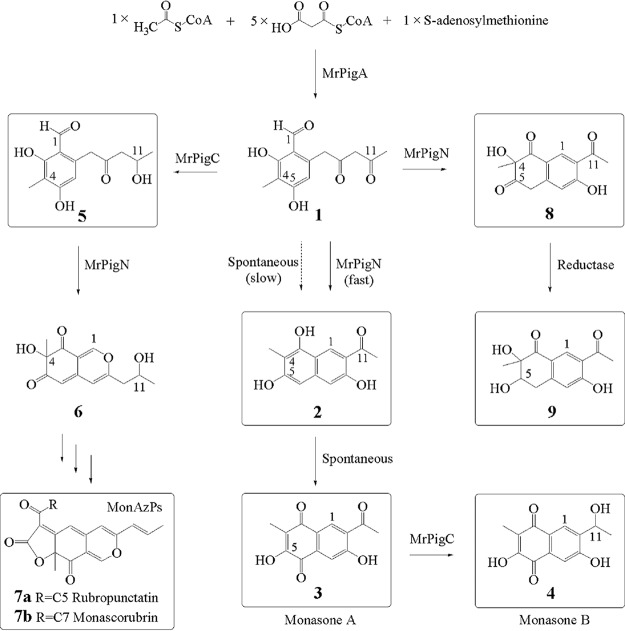
A branching pathway yields MonAzPs and monasone congeners in *M. ruber* M7. The reactive polyketide intermediate **1** gives rise to MonAzPs on one branch (represented by rubropunctatin **7a** and monascorubrin **7b**) and to naphthoquinone congeners on the other branch (represented by trihydroxynaphthalene **2**, monasone A [compound **3**], monasone B [compound **4**], tetralindione **8**, and trihydroxytetralone MA-1 [compound **9**]). The structures of the boxed compounds were elucidated by LC-MS/MS and NMR analysis (data for compounds **2**, **3**, **4**, **8** and **9** are in Table S8 and Fig. S10 [19]).

Our previous studies have indicated that MonAzPs are largely (but not entirely) absent and that trihydroxynaphthalene **2** and monasones **3** and **4** (Fig. 1) are the major products of an *M. ruber* M7 mutant in which *mrpigC*, a gene within the MABGC, was deleted (16–18). This gene encodes the ketoreductase MrPigC that affords benzaldehyde **5** during MonAzP biosynthesis by reducing the C-11 ketone of the unstable intermediate **1**, the putative product of MrPigA, a nonreducing polyketide synthase (nrPKS) (Fig. 1). We proposed that in the absence of MrPigC, intermediate **1** undergoes spontaneous aldol condensation to yield trihydroxynaphthalene **2**, and this derailment product (compound **2**) is subject to spontaneous oxidization to monasone A (compound **3**), which in turn may be fortuitously reduced to monasone B (compound **4**) by *Monascus* enzymes. Therefore, we and others interpreted these results by regarding monasones as incidental shunt products that may only be obtained in substantial amounts by blocking MonAzP biosynthesis (15–17).

To our surprise, we have found that cocultivation of the wild-type *M. ruber* M7 with various bacteria or fungi elicits the biosynthesis of compounds **2**, **3**, and **4** (see Table S3 in reference 19), while the production of MonAzPs remains undisturbed under these culture conditions (Table S4 [19]). All MonAzPs and the monasone congeners **2**, **3**, and **4** should be derived from *M. ruber* M7, since these compounds were not detected upon monoculture of the challenger microorganisms (Fig. S1, Penicillium expansum ATCC 7861 as an example [19]). Furthermore, we found that monasones **3** and **4** exhibit broad-spectrum antibiotic activity against Gram-positive and Gram-negative bacteria and antifungal activity against filamentous fungi, including the monasone-producing *M. ruber* strain itself (Table S5 [19]). Thus, we consider that under some ecologically relevant conditions (such as growth in mixed cultures), these supposed shunt products appear as authentic SMs that may function as allelochemicals against potential competitors of the producing fungi in their natural ecosystem (20).

### A nested subset of coevolving genes within the MABGC correlates with monasone production.

We have confirmed that production of monasones by *M. ruber* M7 requires MrPigA, the nrPKS that assembles intermediate **1**, since monasones **2**, **3**, and **4** were not produced by the Δ*mrpigA* knockout strain in monoculture or under monasone-eliciting cocultivation conditions (Table S3 [19]). Furthermore, bacteria and fungi did not stimulate *M. ruber* M7 to produce monasones when the gene *mrpigB* encoding MrPigB (also known as PigR) (21), the MonAzP cluster-specific positive regulator, was deleted (Table S3 [19]).

**FIG 2:**
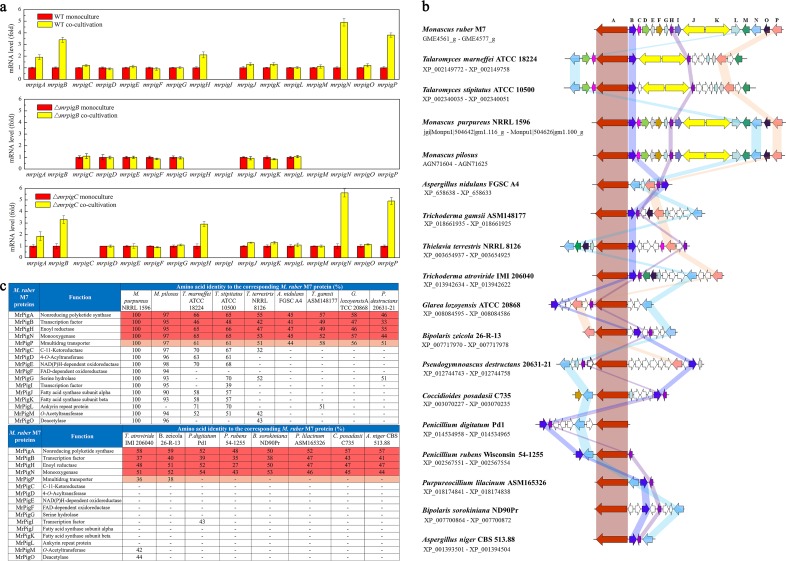
A conserved MrPigB regulon in MABGC-like gene clusters. (a) qRT-PCR analysis of the MABGC genes of the *M. ruber* M7 wild-type (WT), Δ*mrpigB*, and Δ*mrpigC* knockout strains, measured from monoculture or during cocultivation with *Penicillium expansum* ATCC 7861. Gene expression levels from monoculture are taken as the basis of comparison, with the means and standard deviations calculated from measurements from three biological replicates for each strain/cultivation condition shown. (b) MABGC-like gene clusters in filamentous fungal genomes. Arrows with identical colors indicate orthologous genes; white arrows show nonorthologous genes. (c) Proteins encoded by MABGC-related SM gene clusters of various fungi. Proteins highlighted in red are encoded by all MABGC-like gene clusters. Genes encoding orthologues of the MFS transporter MrPigP (highlighted in pink) are present in the majority of the MABGC-like gene clusters.

To further confirm *mrpigB* function, we reevaluated the effects of the deletion of *mrpigB* on the transcription of each gene in the 16-gene MABGC by quantitative reverse transcription-PCR (qRT-PCR) analysis, both under MonAzP-competent monoculture and monasone-eliciting cocultivation conditions (Fig. 2a). During MonAzP-producing monoculture, all MABGC genes were expressed in the wild-type strain, apart from *mrpigI* that encodes a transcription factor whose role in MonAzP biosynthesis is unknown. Unexpectedly, as shown by the Δ*mrpigB* strain, MrPigB controls the expression of only six genes in the MABGC: *mrpigA*, the nrPKS; *mrpigH*, an enoyl reductase; *mrpigM* and *mrpigO*, an acetyltransferase-deacetylase pair; *mrpigN*, a flavin adenine dinucleotide (FAD)-dependent monooxygenase; and *mrpigP*, a major facilitator superfamily (MFS) transporter (Fig. 2b) (15). Furthermore, cocultivation did not restore the expression of these six genes in the Δ*mrpigB* strain, indicating that transcriptional activation of these genes upon elicitation is achieved primarily through MrPigB. This tight control by a transcriptional regulator purportedly governing MonAzP production was unexpected for some of these genes, since among the six genes in the MrPigB regulon, MrPigP had been shown to be unnecessary for MonAzP production, while MrPigH plays only a supplementary role in the biosynthesis of these pigments (15–17).

To obtain an evolutionary perspective on the MrPigB regulon, we analyzed filamentous fungal genomes available in the NCBI GenBank and in the “1000 Fungal Genomes Project” database of the US Department of Energy Joint Genome Institute (http://1000.fungalgenomes.org, accessed 3 October 2019) for the presence of MABGC-related SM biosynthetic gene clusters. Apparent SM clusters with a gene encoding an nrPKS orthologous (>45% identity over the full length of the enzyme) to MrPigA from the MABGC of *M. ruber* M7 were found to exist in the genomes of 17 different fungal species from the genera *Monascus*, *Talaromyces*, *Aspergillus*, *Penicillium*, and *Coccidioides* (Fig. 2b and c). Curiously, genes for orthologues of MrPigB and those for three proteins encoded in the MrPigB regulon (MrPigA, MrPigH, and MrPigN) were seen to be cooccurring and highly conserved in all these gene clusters (Fig. 2b and c). Orthologues of the multidrug transporter MrPigP are found in 11 of the 17 gene clusters (Fig. 2b), while orthologues of the rest of the MonAzP genes, including those encoding MrPigM and MrPigO from the MrPigB regulon of *M. ruber* M7, are only present in the minority of these putative SM biosynthetic gene clusters.

### Monasone biosynthesis is encoded by genes in the MrPigB regulon of *M. ruber* M7.

**FIG 3:**
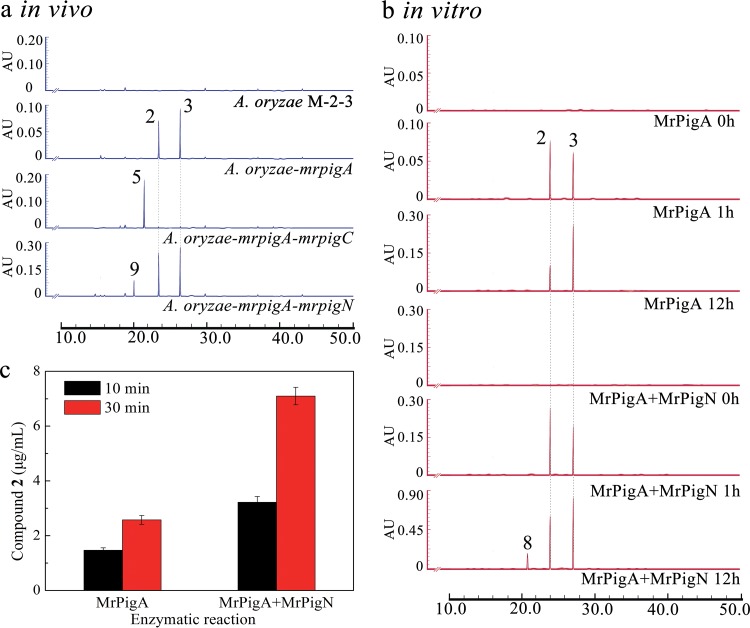
Biosynthesis of monasones during *in vivo* and *vitro* enzymatic reactions with MrPigA and MrPigN. (a) Metabolite profiles (reversed-phase HPLC traces recorded at 280 nm with a photodiode array detector) of fermentation extracts of *A. oryzae* M-2-3 expressing the indicated MABGC genes. (b) *In vitro* enzymatic assays with the indicated purified MABGC enzymes (reversed-phase HPLC traces recorded at 280 nm with a photodiode array detector). (c) Quantification of trihydroxynaphthalene **2** in enzymatic reactions with the indicated enzymes after 10 min or 30 min. Yields of compound **2** are shown in micrograms per milliliter as the means ± standard deviations (SDs) from three independent experiments of three replicates each, *n* = 9. Statistical analysis with Student's *t* test revealed that there was a significant difference between group MrPigA and group MrPigA+MrPigN at *P* < 0.05.

MrPigA was characterized in our previous work by gene knockout as the nrPKS responsible for the production of the polyketide scaffold of MonAzPs (15). MrPigA was also shown to yield large amounts of trihydroxynaphthalene **2** and smaller amounts of monasones **3** and **4** upon heterologous expression in yeast (15). In the present study, MrPigA was heterologously expressed in Aspergillus oryzae M-2-3 to yield compounds **2** and **3** (2.2 and 7.1 mg/liter) (Fig. 1 and 3a). We also observed that purified trihydroxynaphthalene **2** readily autooxidized into monasone A (compound **3**) in aqueous solutions under aerobic conditions (Fig. S2a [19]). Remarkably, coexpression of *mrpigA* and *mrpigN* in *A. oryzae* led to the production of significantly larger amounts of compounds **2** and **3** (7.2 and 18.4 mg/liter) (Fig. 3a). Increased production of monasones was unexpected, as MrPigN had earlier been identified as the enzyme that is responsible for the hydroxylation of C-4 of benzaldehyde **5** during MonAzP biosynthesis (Fig. 1). However, MrPigN had not been implicated in naphthoquinone production until now, and monasones **3** and **4** do not feature a C-4 tertiary alcohol.

To further demonstrate the involvement of MrPigN in monasone biosynthesis, we expressed *mrpigA* and *mrpigN* under the strong promoter P*_amyB_* in *A. oryzae* M-2-3 and purified the corresponding recombinant proteins by sequential ultrafiltration, ammonium sulfate precipitation, and Ni-Sepharose column chromatography to yield 23 mg MrPigA and 42 mg MrPigN from 100 g wet mycelia (Fig. S3 [19]). Production of compound **2** was recapitulated in a reconstituted *in vitro* enzymatic assay with MrPigA from acetyl coenzyme A (acetyl-CoA), malonyl-CoA, NADPH, and *S*-adenosyl methionine as the substrates. Small amounts of the autooxidation product monasone A (compound **3**) were also detected in the reaction. Meanwhile, recombinant MrPigN was shown to efficiently convert benzaldehyde **5** into azanigerone E (compound **6**), as expected (Fig. S2b [19]). Importantly, coupled reactions with MrPigA and MrPigN afforded approximately 100% more product **2** than the reaction with MrPigA alone (Fig. 3b and c). Extended *in vitro* reactions with MrPigA and MrPigN also yielded minor amounts of tetralindione **8**, the C-4 hydroxylated analogue of trihydroxynaphthalene **2** (Fig. 3b). MA-1 (compound **9**) that derives from compound **8** by reduction was also detected in fermentations with *A. oryzae* strains coexpressing *mrpigA* and *mrpigN* (Fig. 3a), just as with recombinant yeast expressing these two genes, or with the Δ*mrpigC* knockout mutant of *M. ruber* M7 (15, 17).

Taken together, these observations indicate that MrPigN not only is involved in MonAzP biosynthesis but also facilitates the formation of trihydroxynaphthalene **2**, the precursor of monasones. To account for this result, we propose that during MonAzP biosynthesis, benzaldehyde **5** is readily hydroxylated by MrPigN to yield azanigerone E (compound **6**) (Fig. 1). However, in the absence of MrPigC, MrPigN may also intercept intermediate **1**, and the binding may assist the folding of this reactive intermediate into a pose conducive to the aldol condensation in the C1-C10 register, thereby increasing the yield of trihydroxynaphthalene **2** and its naphthoquinone derivatives. Importantly, compounds **2**, **3**, and **4** were not accepted as the substrates for C-4 hydroxylation by MrPigN (Fig. S2c [19]), while intermediate **1** may not be a preferred substrate for hydroxylation, as shown by the very small amounts of compound **8** produced in the MrPigA+MrPigN coupled enzymatic assay and the low yield of MA-1 (compound **9**) during the coexpression of the corresponding genes in heterologous hosts.

The genes *mrpigM* and *mrpigO* encode an acetyltransferase-deacetylase pair necessary for MonAzP biosynthesis. Although these genes are part of the MrPigB regulon in *M. ruber* M7, they are not conserved in MABGC-like biosynthetic gene clusters (Fig. 2). Deletion of these genes did not influence monasone congener production in Δ*mrpigM* and Δ*mrpigO* strains upon monoculture or during cocultivation with *P. expansum* ATCC 7861 (Table S3 [19]), indicating that the corresponding enzymes do not participate in monasone biosynthesis.

### Reductive transformations of monasones by MrPigH may contribute to self-resistance.

**FIG 4:**
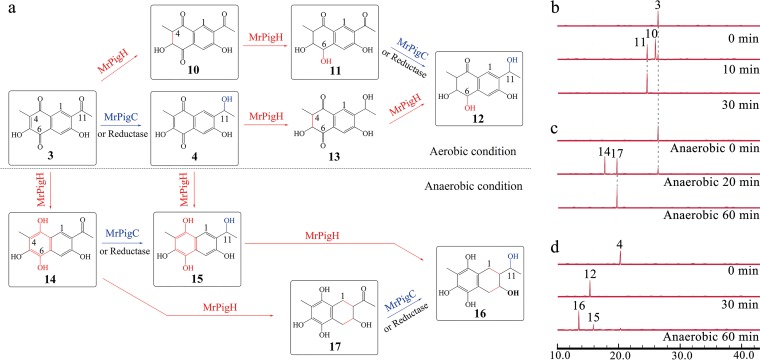
MrPigH-mediated reductive transformation of monasones. (a) Proposed metabolic grid for the enzymatic reduction of monasone A (compound **3**) by MrPigH and MrPigC or a similar ketoreductase under aerobic or anaerobic conditions. The structures of the boxed compounds were elucidated by LC-HRMS/MS and NMR analysis (Table S8 and Fig. S10 [19]). (b) Time course analysis of the reduction of monasone A (compound **3**) into compounds **10** and **11** by recombinant MrPigH under aerobic conditions. (c) Time course analysis of the reduction of monasone A (compound **3**) into compounds **14** and **17** by MrPigH under anaerobic conditions. (d) Reduction of monasone B (compound **4**) by recombinant MrPigH into compound **12** under aerobic conditions and to compound **16** under anaerobic conditions. Reconstituted enzymatic reactions were performed in HEPES buffer (pH 7.0) containing 1.5 mM substrate and 1.5 mM NADPH at 30°C, and metabolites were detected by reversed-phase HPLC at 280 nm with a photodiode array detector.

The MABGC gene *mrpigH* is part of the MrPigB regulon in strain M7, and orthologues of MrPigH are encoded by all of the MABGC-related SM biosynthetic gene clusters detected in databases (Fig. 2). MrPigH was proposed to contribute to, but is not strictly necessary for, the reduction of the C5(2′) double bond in MonAzP intermediates *en route* to the classical yellow MonAzPs monascin and ankaflavin (15, 16). MrPigH is a short-chain dehydrogenase/reductase similar to enol reductases acting on various aromatic hydrocarbon substrates (22). Since monasones **3** and **4** feature enol groups, we wondered whether MrPigH may also be involved in the biosynthesis of naphthoquinone derivatives. Thus, we turned to *in vitro* reconstitution of the MrPigH-catalyzed reaction. The gene encoding MrPigH was expressed in *A. oryzae* M-2-3 under the inducible promoter P*_amyB_*, and 73 mg of MrPigH was purified from 100 g wet mycelia (Fig. S3 [19]). Considering that reductases such as MrPigH may yield different products under aerobic versus anaerobic conditions (23), we conducted *in vitro* assays under both conditions with recombinant MrPigH. When monasone A (compound **3**) was used as the substrate in the presence of NADPH, distinct product pairs were detected under aerobic versus anaerobic conditions (compounds **10** and **11** versus **14** and **17**, respectively) (Fig. 4). The structures of all four products were elucidated by high-resolution mass spectrometry (HRMS) and nuclear magnetic resonance (NMR) (Table S8 and Fig. S10 [19]). We conclude that MrPigH catalyzes a stepwise reduction sequence. Under aerobic conditions, reduction of the C4(5) enol yields the 4,5-dihydronaphthoquinone **10** with a secondary alcohol at C-5, followed by a further reduction to afford the 5,6-diol **11** (Fig. 4a and b). Under anaerobic conditions, monasone A (compound **3**) was converted by MrPigH into the tetrahydroxynaphthalene **14** by reductive aromatization, and further reduced and dearomatized to form the tetrahydroxytetralin **17** (Fig. 4a and c). Formation of tetrahydroxynaphthalene **14** was observed only under anaerobic conditions. Furthermore, while purified compound **14** was stable in the absence of oxygen in aqueous solutions, it was spontaneously oxidized to monasone A in the presence of oxygen (Fig. S4 [19]).

We previously observed the cooccurrence of monasone B (compound **4**) with its congener monasone A (compound **3**) in *M. ruber* M7 and in recombinant yeast and *A. oryzae* strains expressing *mrpigA* (15, 16). To account for the reduction of the carbonyl at C-11 of monasone A (compound **3**), we decided to investigate MrPigC that reduces the analogous carbonyl in the putative nrPKS product **1**. We purified the recombinant enzyme from *A. oryzae* expressing the mrpigC gene (Fig. S3 [19]) and used the clarified mycelial lysate of the Δ*mrpigC* knockout strain of *M. ruber* M7 as a control. To our surprise, both purified MrPigC and the MrPigC-free *M. ruber* M7 protein extract catalyzed the facile conversion of monasone A (compound **3**) to monasone B (compound **4**) (Fig. 4a; Fig. S5a [19]). Similarly, both purified MrPigC and the Δ*mrpigC* lysate efficiently reduced the C-11 carbonyl of trihydroxytetralone **11** to afford compound **12** (Fig. 4a; Fig. S5b [19]), that of tetrahydroxynaphthalene **14** to produce compound **15** (Fig. 4a; Fig. S5c, anaerobic conditions [19]), and that of tetrahydroxytetralin **17** to yield compound **16** (Fig. 4a; Fig. S5d [19]). Compounds **4**, **12**, **15**, and **16** were isolated and characterized (Table S8 and Fig. S10 [19]). These experiments indicate that MrPigC shows considerable substrate promiscuity paired with strict regiospecificity in reducing the C-11 carbonyl of benzaldehyde (compound **1**), naphthoquinone (compound **3**), tetrahydroxynaphthalene (compound **14**), tetrahydroxytetralin (compound **17**), and trihydroxytetralone (compound **11**) scaffolds (Fig. 4a). Apparently, *M. ruber* M7 also expresses another enzyme(s) that is able to carry out this same C-11 reduction, as was seen earlier with the Δ*mrpigC* strain that still produced small amounts of MonAzP congeners (15). Fittingly, the *M. ruber* M7 genome encodes two orthologues of MrPigC (GME804_g and GME2122_g, with 46% and 33% identities to MrPigC, respectively), although the role(s) of these proteins in these reductions would need to be confirmed by further experiments.

We also demonstrated that the recombinant MrPigH enzyme can catalyze enol reduction with monasone B (compound **4**) as the substrate, affording trihydroxytetralone **12** under aerobic conditions through the putative intermediate **13** (Fig. 4a and d). MrPigH is also able to convert monasone B (compound **4**) to tetrahydroxytetralin **16** through intermediate **15** under anaerobic conditions (Fig. 4a and d). All these reactions catalyzed by MrPigH and MrPigC form a complex metabolic grid that transforms monasone A (compound **3**) to trihydroxytetralone **12** through a six-electron reduction sequence under aerobic conditions or to tetrahydroxytetralin **16** through an eight-electron reduction sequence under anaerobic conditions (Fig. 4a).

We have also tried to validate the contributions of MrPigH to monasone transformations *in vivo*. Despite our best efforts, we were unsuccessful in knocking out or silencing the *mrpigH* gene in *M. ruber* M7. Similarly, replacing the promoter of *mrpigH* with an inducible one such as P_adh1_ or P_cbh1_ was also unsuccessful. However, MrPigH-generated compounds **10**, **11**, **12**, and **16** were detected by liquid chromatography-mass spectrometry (LC-MS) in fermentations of wild-type *M. ruber* M7 under monasone-eliciting cocultivation conditions but not in monoculture (Table S6 [19]). Strikingly, the Δ*mrpigC* knockout mutant produced all naphthoquinone congeners (compounds **2** to **4**, **10** to **12**, and **14** to **17**) under cocultivation conditions, presumably due to the additional C-11 ketoreductase activity of the fungus (Table S6 [19]). Next, we coexpressed *mrpigH* with *mrpigA* and *mrpigN* in *A. oryzae* M-2-3. Compared to that in the *A. oryzae* strain expressing *mrpigA* and *mrpigN*, production of trihydroxynaphthalene **2** and monasone A (compound **3**) was drastically reduced (Fig. S6a [19]). However, compounds **4**, **10** to **12**, and **14** to **17** were not detected in the *A. oryzae* production system (Fig. S6 [19]). Feeding any one of the compounds **2** to **4**, **10** to **12**, or **14** to **17** (each at 0.5 mg/liter) to *A. oryzae* M-2-3 showed that compounds **2** and **3** are stable, but compounds **4**, **10** to **12**, and **14** to **17** disappear during incubation with mycelia of this host (Fig. S6 and Table S7 [19]). Thus, naphthoquinone congeners produced by MrPigH from compounds **2** and **3** are readily catabolized by endogenous enzymes of the *Aspergillus* host.

Monasone A (compound **3**) and B (compound **4**) were confirmed to show antifungal and broad-spectrum antibacterial activities, as expected (Table S5 [1, 2, 19]). However, compounds **12** and **16**, the reduced derivatives of monasones A and B, displayed no antifungal or antibiotic activities against the same panel of microorganisms (Table S5 [19]). Intriguingly, while monasones A and B displayed self-toxicity to the producer fungus *M. ruber* M7, compounds **12** and **16** were found not to be growth inhibitory. Considering that the production of MrPigH is coregulated with the enzymes that produce monasones **3** and **4**, these results suggest that the reductive transformations initiated by MrPigH may contribute to the self-resistance of *M. ruber* M7 against these compounds.

### MrPigP is a monasone exporter that also contributes to self-resistance in *M. ruber* M7.

**FIG 5:**
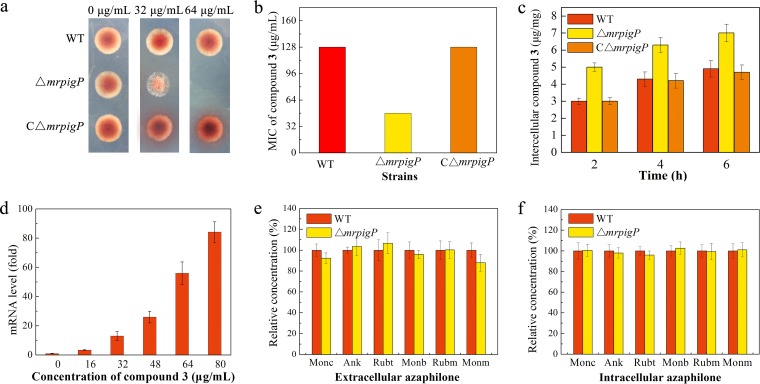
MrPigP is an inducible naphthoquinone transporter. (a) Growth of wild-type (WT) *M. ruber* M7, the Δ*mrpigP* knockout mutant, and the *mrpigC*-complemented knockout strain (CΔ*mrpigP*) on PDA plates containing 32 μg/ml or 64 μg/ml monasone A (compound **3**) at 30°C for 5 days. (b) MICs of the WT, Δ*mrpigP*, and CΔ*mrpigP* strains evaluated after cultivation in PDB at 30°C for 5 days. (c) Flux assay measuring the intracellular concentration of monasone A (compound **3**) in the WT, Δ*mrpigP*, and CΔ*mrpigP* strains after immersion in 32 μg/ml monasone A for 6 h. Statistical analysis using Student's *t* test revealed that there was a significant difference (*P* < 0.05) between group Δ*mrpigP* and either group WT or group CΔ*mrpigP*. Groups WT and CΔ*mrpigP* were not significantly different. (d) Relative transcription levels of the *mrpigP* gene after growth of the wild-type strain on different monasone A (compound **3**) concentrations. The β-actin gene was used as an internal standard to normalize expression levels. Extracellular (e) and intracellular (f) MonAzP concentrations in the WT and Δ*mrpigP* strains. The strains were cultivated in PDB medium at 30°C for 10 days (mid-production phase). MonAzPs measured: Monc, monascin; Ank, ankaflavin; Rubt, rubropunctatin; Monb, monascorubrin; Rubm, rubropunctamine; Monm, monascorubramine. Statistical analysis using Student's *t* test revealed no significant differences between groups WT and Δ*mrpigP* for any of these compounds. Data from all quantitative experiments are shown as the means ± SDs from three independent experiments of three replicates each, *n* = 9.

The MrPigB regulon of *M. ruber* M7 includes the gene *mrpigP* that encodes a major facilitator superfamily (MFS) multidrug transporter. MrPigP is not necessary for MonAzP production, as Δ*mrpigP* knockout strains continue to produce these azaphilone pigments undisturbed (18, 24). Although MrPigP orthologues are not universally conserved in MABGC-like clusters in fungi, we were intrigued by the possibility that MrPigP may be involved in the efflux of naphthoquinone congeners, at least in *M. ruber* M7. To test this hypothesis, we constructed the *mrpigP* gene knockout mutant (Δ*mrpigP*) and the *mrpigP*-complemented knockout strain (CΔ*mrpigP*) (Fig. S7 [19]). Then, we compared the antifungal activities of monasones A and B and their reduced derivatives **12** and **16** against those of the wild-type *M. ruber* M7 and the Δ*mrpigP* and CΔ*mrpigP* strains. The Δ*mrpigP* strain turned out to be markedly more sensitive to monasone A (compound **3**) and monasone B (compound **4**) than the wild type or the complemented strains (Fig. 5a and b and Table S5 [19]) while just as resistant to compounds **12** and **16** as the wild type or the complemented strain.

Next, we performed flux assays with the same compound-strain combinations. Mycelia of wild-type *M. ruber* M7, Δ*mrpigP*, and CΔ*mrpigP* strains were grown in potato dextrose broth (PDB) for 7 days and then transferred to phosphate-buffered saline (PBS) containing monasone A (compound **3**), monasone B (compound **4**), compound **12**, or compound **16**. After 6 h, the intracellular concentration of monasone A or B in the Δ*mrpigP* strain was found to be considerably higher than in the wild type or the CΔ*mrpigP* strain (Fig. 5c; Fig. S8 [19]). However, the intracellular concentrations of compounds **12** and **16** displayed no obvious differences among the wild-type, Δ*mrpigP*, and CΔ*mrpigP* strains under the same conditions.

We also used qRT-PCR to quantify the expression of the *mrpigP* gene when *M. ruber* M7 was grown for 5 days in the presence of different concentrations of monasone A (compound **3**), monasone B (compound **4**), compound **12**, or compound **16**. The results indicated a notable increase in *mrpigP* expression with increasing concentrations of monasone A or monasone B (Fig. 5d; Fig. S9 [19]). Incubation with compound **12** or **16** led to only a weak induction of *mrpigP* expression (Fig. S9 [19]).

Finally, to exclude a determining role of MrPigP in MonAzP export, we compared the intracellular and the extracellular concentrations of representative MonAzPs in the wild-type *M. ruber* M7 and the Δ*mrpigP* strains at mid-production phase (fermentations usually complete in 12 to 15 days). No obvious difference was detected between the two strains (Fig. 5e and f). Taken together, these experiments confirm that MrPigP is not involved in MonAzP biosynthesis or export, as suggested earlier (15–17). Instead, it is a naphthoquinone-inducible and naphthoquinone-specific transporter that contributes to the self-resistance of *M. ruber* M7 to monasone A (compound **3**) and B (compound **4**).

## DISCUSSION

The evolution of the ability to synthesize specialized “secondary” metabolites is considered to have been crucial for the survival and diversification of fungal species (11, 25). *Monascus* spp. and other filamentous fungi produce naphthoquinones such as monasones A and B that may inhibit or kill other microorganisms in the natural ecosystem (15, 16), thereby providing an adaptive advantage to their producers. Inevitably, naphthoquinone-producing fungi also must evolve self-protection mechanisms against their own SMs to avoid suicide. These mechanisms may involve active efflux, which also helps to “broadcast” these antimicrobial agents into the environment, and modification of residual SMs inside the producer cells to reduce their toxicity (11, 26).

**FIG 6:**
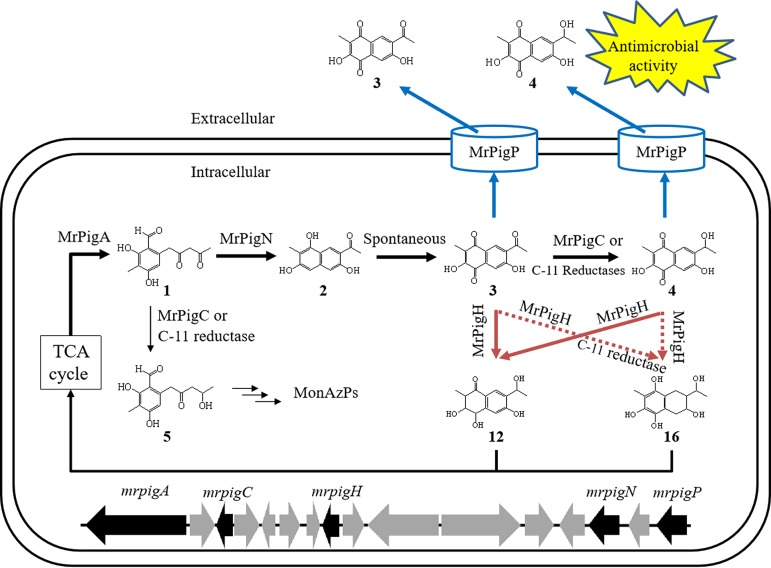
Model for monasone biosynthesis, export, and reductive detoxification in *M. ruber* M7. Thick black arrows show steps in monasone biosynthesis and recycling; thin black arrows indicate steps in MonAzP biosynthesis; blue arrows represent monasone export; dotted and solid red arrows show aerobic and anaerobic monasones detoxification steps, respectively; black block arrows indicate genes for monasone and/or MonAzP biosynthesis and resistance; gray block arrows show genes involved only in MonAzP biosynthesis.

Based on the results described in the present study, we propose a model for the biosynthesis and detoxification of the antifungal and antibiotic monasones in *M. ruber* M7 (Fig. 6). MrPigA and MrPigN, encoded by genes in the MABGC regulated by MrPigB, catalyze the biosynthesis of monasone A (compound **3**) and monasone B (compound **4**) via the intermediate trihydroxynaphthalene **2**. MrPigP, a multidrug transporter also encoded by the MrPigB regulon of the MABGC, exports these toxic metabolites out of the mycelia, thereby protecting *M. ruber* M7 and inhibiting competing microorganisms in the environment. Meanwhile, residual monasones A and B inside the cells may be reduced to nontoxic compounds such as trihydroxytetralone **12** and tetrahydroxytetralin **16** by MrPigH also encoded by the MrPigB regulon, with the help of MrPigC from MonAzP biosynthesis or other reductases of *M. ruber* M7. Presumably, compounds **12** and **16** may be recycled through the tricarboxylic acid (TCA) cycle following aromatic ring cleavage (27).

Our findings reveal the existence of a group of conserved, coevolving, and coregulated genes that form a monasone subcluster nested within the MABGC (Fig. 2). The MABGC has previously been considered to have evolved to produce azaphilone pigments (MonAzPs) and to yield metabolites such as trihydroxynaphthalene **2** and monasone A (compound **3**) only as shunt products upon the blockade of the normal course of MonAzP assembly (15, 16). However, we believe that this view of monasones as mere derailment products is unjustified. First, monasone production is elicited upon challenge by various microorganisms (Table S3 [19]), and these compounds show antifungal and antibacterial activities (Table S5 [19]). Second, the monasone subcluster encodes not only biosynthetic enzymes but also a transcriptional regulator and proteins responsible for two distinct monasone resistance mechanisms (Fig. 2). Third, orthologues of *mrpigA* whose nrPKS products generate a common polyketide intermediate for naphthoquinone congeners and MonAzPs are much more widely associated with genes that are involved in naphthoquinone biosynthesis, resistance, and regulation than with those that are specific for MonAzP production (Fig. 2). In fact, several genes in MABGCs were found not to play an essential role in azaphilone biosynthesis (15, 16). Consequently, we propose that naphthoquinones are bona fide SMs whose biosynthetic mechanisms served as the foundation for the evolution of MonAzP biosynthesis in a group of naphthoquinone-producing fungi. Thus, MonAzP biosynthesis sequesters the pluripotent and unstable polyketide intermediate **1** to afford azaphilones, metabolites that occupy a distinct segment of the SM structural space compared to that by the ancestral naphthoquinones. Nevertheless, the two pathways remain intertwined not only by the corresponding genes being physically colocated on the chromosome but also by several enzymes playing double duty in the two pathways.

MrPigC and its functional equivalent(s) in *M. ruber* M7 reduce the C-11 carbonyl of intermediate **1** *en route* to MonAzPs, and MrPigH contributes to (although is not strictly necessary for) the reduction of enolic intermediates to afford yellow MonAzPs such as monascin and ankaflavin (15). However, the same enzymes also take part in the reduction of the analogous carbons in monasone derivatives to eliminate the self-toxicity of residual intracellular naphthoquinones (Fig. 4). In contrast, MrPigN is also involved in both pathways but in different capacities. For MonAzP biosynthesis, MrPigN hydroxylates the C-4 position of benzaldehyde **5**, while for naphthoquinone biosynthesis, the same enzyme facilitates C1/C10 aldol condensation in intermediate **1** to afford monasone A (compound **3**), with only minor amounts of C-4 hydroxylated analogues produced (Fig. 1).

Another level of connection is provided by the cluster-specific regulator MrPigB that affects both pathways through controlling the expression of MrPigA (an nrPKS). This regulator also controls most of the identified genes of the naphthoquinone pathway (*mrpigN*, *mrpigH*, and *mrpigP*), while only a small subset of the MonAzP genes belong to the MrPigB regulon (Fig. 2). Similar composite “superclusters” responsible for the production of more than one SM each were also observed recently in other fungi such as Aspergillus terreus, Aspergillus nidulans, and Aspergillus fumigatus (28–30). These superclusters have evolved by expansion, merger, and diversification of ancestral gene clusters responsible for the production of archetypal SMs. Just as with the monasone-MonAzP biosynthetic gene supercluster, certain genes of these superclusters are involved in the biosynthesis of more than one SM, while other genes only take part in the biogenesis of one particular SM (28, 30). This mosaic nature of superclusters makes bioinformatic prediction of gene functions problematic, but coupled with functional analysis, it will help to illuminate the evolutionary paths that fungal SM biosynthesis has traveled.

Secondary metabolite natural products are often produced as allelochemicals, and as such, their production is often triggered by interactions of the producer with other microorganisms or plant and animal hosts (25). Cocultivation of microorganisms has also been applied in the laboratory to elicit the biosynthesis of novel SMs or to promote the production of known SMs (31). Our reassessment of monasones as genuine SMs with ecological roles independent of those of MonAzPs was prompted by the observation that cocultivation of *M. ruber* M7 with other microorganisms stimulates the production of these naphthoquinones but not that of the azaphilones (Tables S3 and S4 [19]). Together with their antibacterial and antifungal activities, these results suggest that the biosynthesis of monasones is an ecological fitness trait that helps *M. ruber* M7 defend its niche (32). At the same time, production of monasones may be further promoted by optimizing cocultivation parameters by varying the microbial challengers and the interaction modes.

Understanding the mechanisms by which microorganisms that produce antimicrobial agents avoid suicide is crucial for developing new anti-infective drugs that are less susceptible to, and less prone to provoke, resistance in targeted pathogens and nontarget microorganisms of the host microbiome (33, 34). Resistance to antimicrobial agents may involve exclusion or inactivation of the compound, alteration of the target, and phenotypic resistance. Inactivation of toxic (naphtho)quinones by reductases or dehydrogenases has been reported in some microorganisms (22, 35). Our results show that the monasone producer *M. ruber* M7 may apply two-tiered self-defense by using the naphthoquinone-specific and naphthoquinone-inducible transporter MrPigP to expel monasones A and B from the mycelia and may utilize MrPigH and MrPigC to reduce residual intracellular monasones to compounds **12** and **16** that show no notable antimicrobial activity (Fig. 5).

### Conclusions.

Our work provides an example for the existence of mosaic-like superclusters in fungi. These superclusters are responsible for the biosynthesis of more than one SM with highly divergent structures and encode regulators, resistance determinants, and biosynthetic enzymes that may be shared by the constituent pathways or may be relevant only for an individual pathway. Common core enzymes encoded by these superclusters may yield malleable pluripotent intermediates that are further processed by promiscuous tailoring enzymes into structurally and functionally distinct products. Such SM biosynthetic and genomic parsimony provides important clues for the evolution of SM diversity in microorganisms. Functional characterization of the MABGC supercluster also delineates a two-tiered resistance mechanism against naphthoquinones that may transfer to pathogenic microorganisms or emerge in targeted mammalian cells.

## MATERIALS AND METHODS

### Strains and plasmids.

Aspergillus oryzae strain M-2-3, an arginine auxotroph, was obtained from Colin Lazarus (University of Bristol, UK). Monascus ruber strain M7 was previously described (15). Plasmids pEYA and pTAYAGSarg3P were propagated in Escherichia coli TOP10 (Invitrogen) as the host. Gateway destination vectors were propagated in E. coli *ccdB* Survival 2 T1R cells (Invitrogen). Recombinant *M. ruber* and *A. oryzae* strains are listed in Table S1 (19). Primers used in plasmid construction are listed in Table S2 (19).

### Media and cultivation.

E. coli was grown at 37°C in LB supplemented with the appropriate antibiotic. *A. oryzae* strains were routinely cultivated at 28°C in CMP (3.5% Czapek Dox broth, 2% maltose, 1% Polypepton) for 5 days with shaking at 200 rpm and were inoculated (1% [vol/vol]) from a preculture (a 100-ml overnight culture inoculated with 5 × 10^7^ conidia/ml). *M. ruber* strains were cultured in PDB medium at 30°C with shaking at 200 rpm, and the mycelia were harvested after 12 to 15 days of cultivation. For cocultivation, *M. ruber* strains were first grown as a monoculture in 100 ml PDB at 30°C for 3 to 4 days with shaking at 200 rpm, and then 5 to 10 ml of a freshly prepared culture of the challenger fungi or bacteria was added. The coculture was further incubated at 30°C with shaking at 200 rpm for an additional 3 to 5 days. Challenger fungal cultures were prepared by culturing the appropriate strains in 100 ml PDB medium at 28°C for 2 to 4 days with shaking at 120 rpm. Mycelial pellets were dispersed by vortexing and resuspended in 100 ml fresh PDB. Staphylococcus aureus strains were grown in LB medium at 37°C for 24 h with shaking at 200 rpm.

### RNA extraction, cDNA synthesis, and qRT-PCR.

Total RNA was extracted with TRIzol (Qiagen) from mycelial samples that were prefrozen and ground to a fine powder. The resulting RNA samples were checked for purity (optical density ratio at 260 and 290 nm [OD_260/280_] of 1.8 to 2.2) and integrity (observation of two sharp bands for the ribosomal RNAs [rRNA] of the large and the small ribosomal subunits, with the intensity of the larger band approximately twice that of the smaller band). cDNA synthesis was performed in 20-μl reaction mixtures containing approximately 1 μg of total RNA, using the PrimeScript RT master mix (TaKaRa) according to the instructions of the manufacturer. qRT-PCR was performed using 10-μl reaction mixtures containing 5.0 μl SYBR Premix *Ex Taq* II (1×, TaKaRa), 0.4 μl of each primer (400 nM each), 0.4 μl ROX reference dye, and 1.0 μl of cDNA sample. A two-step thermal profile (step 1, 95°C for 10 s, and step 2, 40 cycles of 95°C for 3 s and 60°C for 25 s) was used on a 7500 Fast real-time PCR system (Applied Biosystems). Results were analyzed using the StepOne software (version 2.0; Applied Biosystems). The cycle number at which the fluorescence passed the cycle threshold (*C_T_*) was used for the quantitation of the expression level. Relative expression levels for each target cDNA were obtained by the 2^−ΔΔ^*^CT^* method via normalization to β-actin (GenBank accession no. AJ417880), using the formula 2^−(^*^CT^* ^target −^ *^CT^* ^actin)^. Controls with no added template were used to exclude the possibility that primer-dimer formation interfered with the calculations.

### Expression of biosynthetic genes in *A. oryzae*.

The intron-free *mrpigA* polyketide synthase gene was amplified from *M. ruber* M7 cDNA as three overlapping fragments using primer pairs *mrpigA*-1-F/*mrpigA*-1-R, *mrpigA*-2-F/*mrpigA*-2-R, and *mrpigA*-3-F/*mrpig*A-3-R (Table S2 [19]). The resulting fragments were reassembled in the pEYA vector by homologous recombination in Saccharomyces cerevisiae BY4741. The cloned *mrpigA* gene was then transferred to pTAYAGSarg3P by Gateway LR recombination (Invitrogen) to create pTAYAGSarg3P-*mrpigA*. In this vector, the *mrpigA* gene was under the control of the P*_amyB_* promoter and the T*_amyB_* terminator. Genes encoding tailoring enzymes were amplified from *M. ruber* M7 cDNA using primers (Table S2 [19]) that overlap the 3′ end of the appropriate promoter (P*_adh_* or P*_eno_*) or the 5′ end of the corresponding terminator (T*_adh_* or T*_eno_*), respectively. The PCR products were cloned into pTAYAGSarg3P-*mrpigA* using the HiEff Clone One Step PCR cloning kit (Yeasen). After transformation of *A. oryzae* M-2-3 with the recombinant plasmids (Table S1 [19]), three to five independent isolates were evaluated for metabolite production for each recombinant strain.

### Protein extraction and purification.

Genes encoding biosynthetic enzymes with an added N-terminal 6×His tag were cloned into the pTAYAGSarg3P vector and placed under the control of the P*_amyB_* promoter and the T*_amyB_* terminator. Plasmids were transformed into *A. oryzae* M-2-3 (Table S1 [19]). Recombinant *A. oryzae* strains were cultivated in CMP medium for 2 days with shaking at 200 rpm at 28°C and induced with 0.5% (wt/vol) maltose every day during continued growth for 3 days with shaking at 200 rpm at 28°C. Protein was purified as previous work reported with modifications (3). The mycelia were harvested by centrifugation and disrupted by high-pressure homogenization with a French press in a buffer containing 50 mM sodium phosphate (pH 7.0). Crude MrPigA protein extracts were filtered using a 0.22-μm cellulose nitrate membrane (Millipore) and then ultrafiltered (Millipore) to remove proteins with molecular weights less than 100 kDa. Crude protein extracts with MrPigC, MrPigH, or MrPigN were also clarified using a 0.22-μm cellulose nitrate membrane (Millipore), but the flowthrough fraction was collected during ultrafiltration (100 kDa cutoff; Millipore). The ultrafiltered protein extracts were precipitated with ammonium sulfate (50% to 90%) during overnight incubation at 4°C and then centrifuged, and the pellet was solubilized in 100 mM Tris buffer (pH 7.0). The resulting protein solution was clarified again by centrifugation, and the His-tagged enzymes were purified via Ni affinity chromatography (Thermo) according to the instructions of the manufacturer. The eluate was dialyzed against 20 mM HEPES (pH 7.0) with 50 mM NaCl and concentrated to greater than 1.0 mg/ml using a centrifugal concentrator (Millipore). Protein concentrations were determined using the Bradford assay with bovine serum albumin (BSA) as the standard.

### Protein deglycosylation.

Five microliters of the purified protein sample (5 to 10 μg protein), 1.0 μl 10× glycoprotein denaturing buffer, and 4.0 μl water were mixed and then heated at 100°C for 10 min, followed by the addition of 2 μl 10× GlycoBuffer, 6.5 μl H_2_O, and 1.5 μl Endo H (NEB). The reaction mixtures were incubated at 37°C for 1 h.

### *In vitro* enzymatic reactions.

In general, substrates (1.5 mM each, final concentration) were dissolved in 150 μl HEPES buffer (50 mM, pH 7.0), and the reaction was started by adding 50 μl of the enzyme solution (10 μM, final concentration). Glycosylated enzymes were used for the reactions, as deglycosylated enzymes showed no improvements in their activities. After incubation at 30°C with shaking at 100 rpm for 30 min, the reaction was stopped by addition of 25 μl H_2_SO_4_ (1.0 M). Cosubstrates used for the MrPigA reaction were as follows (final concentrations): malonyl-CoA (10.0 mM), acetyl-CoA (3.0 mM), *S*-adenosyl methionine (SAM; 3.0 mM), NADPH (3.0 mM), and NADP (sodium salt, 3 mM). Reactions with MrPigA and MrPigN used cosubstrates as above. Reactions with MrPigC used NADPH (10 mM) as the cosubstrate. Enzymatic reactions with total protein extracts from *M. ruber* Δ*mrpigC* were performed as with MrPigC but with MrPigC replaced by the clarified lysate of the strain (50 μl). Reactions with MrPigH used NADPH (10 mM) as the cosubstrate and were conducted under aerobic and anaerobic conditions. For anaerobic reactions, nitrogen was bubbled through the buffer solution for 1 h after degassing under reduced pressure. The reaction mixture was carefully flushed with nitrogen and stirred for 30 min under a nitrogen atmosphere. For all reactions, the remaining substrate and the reaction product(s) were extracted with 200 μl of a mixture of hexane and ethyl acetate (7:3) three times, and the pooled organic phase was dried under a nitrogen stream, dissolved in 200 μl high-pressure liquid chromatography (HPLC)-grade methanol and analyzed by HPLC and NMR. Quantitative assays were repeated three times with three technical replicates each (*n* = 9). Qualitative assays were performed using three biological replicates.

### Extraction of metabolites.

Mycelia of *M. ruber* strains were collected after cultivation for 12 to 14 days, while *A. oryzae* mycelia were harvested at day 5. Wet mycelia (100 g) were disrupted in a French press by high-pressure homogenization, acidified with 37% HCl to a pH less than 4.0, and extracted three times with 50 ml each of a mixture of hexane and ethyl acetate (7:3) by vigorous shaking for 1 h. The pooled organic fraction was dried on a rotary evaporator, and the crude extract was dissolved in 5 ml HPLC-grade methanol.

### Isolation of compounds.

Compounds were isolated by HPLC on a Shimadzu autopurification system equipped with a supplementary L-10AP autosampler, an LC-6AD pump system, an SPD-M20A detector, and a Shim-pack C_18_ column (5.0 μm, 20 mm by 250 mm). The following gradient profile of solvents A and B was used at a column temperature of 30°C and at a flow rate of 20 ml/min: 0 to 5 min, 60% A; 5 to 25 min, 60% to 5% A; 25 to 30 min, 5% A; 30 to 35 min, 5% to 35% A; and 35 to 40 min, 50% A. The mobile phase solvent A was HPLC-grade water, and solvent B was HPLC-grade acetonitrile, both containing 0.1% formic acid. Detected peaks were collected in glass test tubes. The purified fractions were freeze-dried to completely remove the solvent. The purified compounds were subjected to HRMS/MS and NMR analyses.

### HPLC, HRMS, and NMR analyses.

Analysis for metabolites was carried out using a Shimadzu HPLC equipped with an SPD-M20A photodiode array detector and a supplementary L-20A autosampler and managed by a LabSolutions LC Workstation (Shimadzu). The analytes were separated on an Inertsil ODS-3 C_18_ column (5.0 μm, 4.6 mm by 250 mm; Shimadzu) operating at 30°C, and compounds were detected at 280 nm. The mobile phase solvents A and B were water and acetonitrile, both containing 0.05% phosphoric acid. Gradient elution at a flow rate of 0.8 ml/min was performed as follows: 0 to 25 min, 85% to 30% A; 25 to 30 min, 30% to 5% A; 30 to 31 min, 5% to 85% A; 31 to 35 min, 85% A. High-resolution mass spectrometry and tandem mass spectrometry were conducted on a Thermo Q Exactive Plus. ^1^H and ^13^C NMR was conducted on a Varian Mercury Plus 400 NMR spectrometer. The compounds **5**, **7a**, and **7b** were characterized in our previous work (15).

### Antimicrobial susceptibility assays.

The MICs of selected compounds against fungal and bacterial strains were determined using the broth microdilution method. Conidial suspensions of fungi were diluted to a final inoculum concentration of 1.0 × 10^5^ to 5.0 × 10^5^ spores/ml and dispensed into microdilution wells. Bacteria were grown on LB medium overnight at 37°C to an OD_610_ of 1.0 to 1.2 and inoculated (10%) into microdilution wells with fresh LB medium. The final concentrations of the test compounds were 0 to 256 μg/ml. For fungi, the inoculated microdilution trays were incubated at 30°C without shaking, and OD_610_ was measured on days 5 and 7. For bacteria, the inoculated microdilution trays were incubated at 37°C with shaking at 120 rpm, and OD_610_ was measured at 24 h. The MIC endpoint was defined as the lowest concentration that led to complete inhibition of growth. Test compounds were purified by semipreparative HPLC and analyzed by HPLC to show a purity of >95%. Assays were repeated three times with three technical replicates each (*n* = 9).

### Compound biotransformation with resting cells.

*A. oryzae* M-2-3 was cultivated at 30°C in PDB medium with shaking at 200 rpm for 7 days. Compounds (each at 0.5 mg/liter) were added to mycelial pellets suspended in distilled water and incubated at 30°C with shaking at 200 rpm. After incubation, the mycelia were processed by high-pressure homogenization on a GEA Panda2K NS1001L (GEA). The intracellular and extracellular test compounds were extracted by hexane and ethyl acetate (7:3) and then analyzed by HPLC and LC-MS.

### Flux assays.

*M. ruber* M7, Δ*mrpigP*, and CΔ*mrpigP* strains were cultured at 30°C in 30 ml PDB medium in 250-ml flasks with shaking at 200 rpm for 7 days. The mycelia were dispersed by gentle vortexing with the help of glass beads for 5 min and incubated in PBS (50 mM, pH 5.5) containing the test compound (32 μg/ml, final concentration) at 30°C for 6 h with gentle mixing on a rotating table. The mycelia were washed with PBS (50 mM, pH 5.5) at least three times until the test compound was not detected in the eluent. The resulting mycelia were disrupted by high-pressure homogenization on a GEA Panda2K NS1001L (GEA). The intracellular test compounds were extracted every 2 h by hexane and ethyl acetate (7:3) and then analyzed by HPLC. Assays were repeated three times with three technical replicates each (*n* = 9).

### Gene knockout in *M. ruber* M7.

Targeted gene knockout and complementation of *mrpigP* in *M. ruber* M7 was performed as described in our previous work (15), and validation of the strains is shown in Fig. S7 (19).

### Data availability.

Sequences of the genes mentioned in this article are available in GenBank (15).
